# Lymphoid enhancer binding factor-1 (LEF1) expression as a prognostic factor in adult acute promyelocytic leukemia

**DOI:** 10.18632/oncotarget.1619

**Published:** 2013-12-19

**Authors:** Francesco Albano, Antonella Zagaria, Luisa Anelli, Paola Orsini, Crescenzio Francesco Minervini, Luciana Impera, Paola Casieri, Nicoletta Coccaro, Giuseppina Tota, Claudia Brunetti, Angela Minervini, Domenico Pastore, Paola Carluccio, Anna Mestice, Angelo Cellamare, Giorgina Specchia

**Affiliations:** ^1^ Department of Emergency and Organ Transplantation (D.E.T.O.), Hematology Section, University of Bari, Bari, Italy

**Keywords:** acute promyelocytic leukemia, LEF1, prognosis, outcome

## Abstract

Lymphoid enhancer-binding factor 1 (*LEF1*) is a downstream effector of the Wnt/ β-catenin signaling pathway. High *LEF1* expression has been reported as a prognostic marker in hematologic malignancies. We evaluated the prognostic significance of *LEF1* expression in 78 adult acute promyelocytic leukemia (APL) patients. APL samples were dichotomized at the median value and divided into: LEF1^low^ and LEF1^high^. LEF1^high^ patients had lower WBC counts at baseline and were less likely to carry a *FLT3* -ITD than LEF1^low^ patients. Early death occurred only in the LEF1^low^ group. Moreover, LEF1^low^ expression was associated with a high Sanz score. Survival analysis of 61 APL patients < 60 years revealed that the LEF1^high^ group had a significantly longer overall survival (OS). Cox analysis for OS confirmed only *LEF1* expression as an independent prognostic factor. Of the 17 patients over the age of 60, those in the LEF1^high^ group showed a higher median survival. In silico analysis identified 9 differentially expressed, up-modulated genes associated with a high expression of *LEF1*; the majority of these genes is involved in the regulation of apoptosis. Our study provides evidence that *LEF1* expression is an independent prognostic factor in APL, and could be used in patients risk stratification.

## INTRODUCTION

Acute promyelocytic leukemia (APL) is a distinct subtype of acute myeloid leukemia (AML) accounting for about 5% of all cases. APL is characterized by abnormal promyelocytes infiltrating bone marrow and other hematopoietic organs, and t(15;17) translocation leading to *PML-RARα* fusion gene [1]. Induction treatment of APL combining all-trans retinoic acid (ATRA) with anthracycline-based chemotherapy induces a complete remission rate of 90–95% and a cure rate of more than 80% [2,3]. However, improving the relapse rate and incidence of early death may pose the greatest challenge for the future management of APL. The current risk-stratification system for APL is based only on the white-cell and platelet count [4], although these parameters could not be confirmed in the German AMLCG trial for younger patients [5]. New molecular biomarkers may help to make a better risk stratification of APL patients and to identify those with a poorer prognosis.

Lymphoid enhancer-binding factor 1 (*LEF1*) is a downstream effector of the Wnt/β-catenin signaling pathway, which controls cell growth and differentiation [6]. Dysregulation of *LEF1* expression may result in several disease patterns, as the Wnt signaling plays a pivotal role in development and cancerogenesis and also controls self-renewal, proliferation and differentiation of many types of stem cells [7]. However, specific functions of *LEF1* apart from Wnt signaling have also been reported [8], suggesting that this factor may have a more complex role. In fact, in human CD34^+^ hematopoietic progenitor cells, inhibition of *LEF1* but not of β-catenin, impaired proliferation and apoptosis mechanisms of this cell population, supporting the hypothesis of a β-catenin–independent function of *LEF1* in early human myelopoiesis [9]. Recent data in murine model demonstrated that *LEF1* is an important factor for hematopoietic stem and progenitor function and that its stem cell regulatory role depends on its DNA binding ability [10].

In normal human hematopoiesis, *LEF1* plays a pivotal role not only in the development of B- and T-lymphocytes but also in granulopoiesis. In fact, in healthy individuals *LEF1* mRNA levels reached a maximum at the promyelocytic stage of differentiation and declined during the last steps of granulocyte maturation [11]. Recently, deregulated *LEF1* expression, as a mediator of the Wnt pathway, has been implicated in leukemic transformation [12]. High *LEF1* expression has been reported as a favorable prognostic marker in cytogenetically normal AML [13], whereas it is associated with poor prognosis in adult B precursor acute lymphoblastic leukemia [14] and in chronic lymphocytic leukemia [15,16]. Moreover, a marked downregulation of *LEF1* has been associated with disease progression in myelodysplastic syndromes [17]. By contrast, no studies of the prognostic value of *LEF1* expression in adult de novo APL have yet been reported. The *PML-RARα* fusion gene encodes an aberrant transcription factor that shares target genes associated with Wnt signaling [18]. Given the functional role of *LEF1* in hematopoiesis and its putative prognostic impact on several hematological malignancies, we evaluated the prognostic significance of *LEF1* expression in adult de novo APL.

## RESULTS

### *LEF1* expression and pretreatment patient characteristics

**Figure 1:**
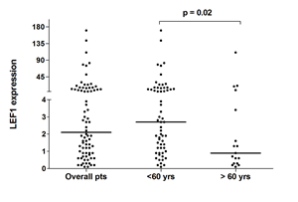
qRT-PCR *LEF1* expression in APL patients Expression of *LEF1* in the overall cohort and in the two groups of patients younger than 60 and older than 60 years. Each dot represents a patient. The lines indicate the median for each group.

The clinical and biological characteristics of the patients included in the study are listed in Table 1. Patients with LEF1^high^ expression had lower white blood cell (WBC) counts at baseline (1.8 vs 12.0 x10^9^/L; p < 0.0001), and were less likely to carry a *FLT3*-ITD than LEF1^low^ patients (12.8% vs 35.9%, respectively, p = 0.02). The association between LEF1^low^ and the presence of *FLT3*-ITD was also confirmed when the 11 (14.1%) patients with *FLT3*-TKD were included among patients with *FLT3* mutations (p = 0.03) or, on the contrary, in the group of *FLT3* wild type patients, as compared to those bearing *FLT3*-ITD (p = 0.03). Early death occurred in 9 (23%) cases in the LEF1^low^ group versus no case in the LEF1^high^ group (OR = 0.04; p= 0.002). Using the PETHEMA relapse risk criteria [16], there were 24 (31%), 40 (51%), and 14 (18%) patients with high, intermediate and low-risk relapse, respectively. LEF1^low^ expression was associated with a higher frequency of a high relapse risk score (53.9% vs 7.7%, OR=0.07; p < 0.0001). The LEF1^high^ group showed a trend toward a statistically significant association with a lower median age (p = 0.08). This trend was confirmed by a statistically significant difference (p = 0.02) when comparing the *LEF1* median expression value in patients aged < 60 and > 60 years (Figure 1). No significant differences were observed regarding CD34, CD2, CD56, bcr3 positivity and *LEF1* gene expression.

**Table 1:** Clinical and molecular features of LEF1 expression in APL patients

	LEF^high^ (n = 39)	LEF1^low^ (n = 39)	*P*
Sex M/F	18/21	19/20	1
Median age, y.rs (range)	44 (16-75)	50 (20-88)	0.08
Median WBC, 10^9^/L (range)	1.8 (0.5-36.5)	12.0 (0.6-147.0)	< 0.0001
Median PLT, 10^9^/L (range)	22.0 (4.0-464.0)	22 (5.0-85.0)	0.4
M3/M3v	38/1	36/3	0.6
Sanz score Low-Intermediate (%) High (%)	36 (92.3%) 3 (7.7%)	18 (46.1%) 21 (53.9%)	< 0.0001
FLT3 mutation status ITD WT TDK	5 (12.8%) 28 (71.8%) 6 (15.4%)	14 (35.9%) 19 (48.7%) 6 (15.4%)	0.02
bcr3/bcr1-2 fusion transcript	19/20	16/23	0.6
CD34 +/−	13/26	9/30	0.4
CD2 +/−	7/32	11/28	0.4
CD56 +/−	3/33	3/27	1
Early death (%)	0	9 (23%)	0.002
Relapse (%)	8 (20.5%)	6 (15.3%)	0.7

### *LEF1* expression and outcome

**Figure 2:**
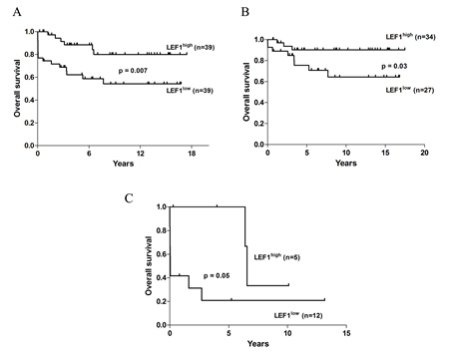
OS analysis of APL patients according to the *LEF1* expression value (A) OS of the entire cohort of APL patients. (B) OS analysis of patients aged younger than 60 years. (C) OS of patients aged older than 60 years.

Among the 78 patients included in the study, the probability of remaining alive after 6 years was 88.4% (95% CI, 77.7%-99.1%) in the LEF1^high^ versus 58.7 % (95% CI, 42.4%-75.1%) in the LEF1^low^ (p=0.007) group (Figure 2A). On the other hand, no differences between the two groups were observed in terms of RFS and CIR (Supplementary Figure S1A). We performed multivariate analyses to determine the prognostic significance of *LEF1* expression after adjusting for the impact of other known risk factors. Cox analysis was performed for hazard OS: among all tested factors (age, relapse risk grade, *FLT3* mutational status, *LEF1* expression) LEF1^high^ expression had an independent prognostic value (HR = 3.4; 95% CI, 1.0-10.5, p=0.03), together with *FLT3*-ITD (HR = 3.9; 95% CI, 1.2-11.8, p=0.01) and age > 60 y.rs (HR = 6.6; 95% CI, 2.7-16.2, p<0.0001) (Table 2). The recurrence rate in our series was 20%; relapsed patients were distributed equally in the two groups (6 in the LEF1^low^ and 8 in the LEF1^high^ group). Survival analysis of 61 (78%) APL patients < 60 years revealed that the LEF1^high^ group again had a significantly longer OS (p = 0.03) (Figure 2B), whereas no differences were observed between the two groups in terms of RFS and CIR (Supplementary Figure S1B). Cox analysis for OS confirmed only LEF1^high^ expression as an independent prognostic factor (HR=5.4; 95% CI, 1.0−27.5, p =0.04) (Table 2). Among the 17 (22%) patients over the age of 60 years, those with LEF1^high^ expression showed a higher median survival (6.5 years vs 0.04 years in the LEF1^low^ group, p = 0.05) (Figure 2C). Cox analysis showed no difference in terms of OS between the two groups (Table 3). RFS and CIR analysis were not performed in this subgroup because of low number of patients obtaining CR.

**Table 2:** Multivariate analyses according to the Cox proportional hazards model Results obtained in all 78 APL patients included in the study (top), and in patients younger than 60 years (bottom). HR indicates hazard ratio; CI, confidence interval.

Overall cohort		
	HR (95% CI)	*P*
Variable		
Age, <60 vs > 60 years	6.58 (2.6-16.1)	<0.0001
FLT3, ITD vs WT + TDK	3.9 (1.2-11.8)	0.01
LEF1 expression, LEF1^high^ vs LEF1^low^	3.3 (1.0-10.5)	0.03
Sanz score, L-I vs H	1.7 (0.5−5.0)	0.3
Young (<60 years) patients		
	HR (95% CI)	P
Variable		
LEF1 expression, LEF1^high^ vs LEF1^low^	5.4 (1.0-27.0)	0.04
FLT3, ITD vs WT + TDK	0.4 (0.08-2.0)	0.2
Sanz's score, L-I vs H	1.0 (0.2−3.8)	0.9

**Table 3:** Results of Cox analysis in the older than 60 years APL patients included in the study HR indicates hazard ratio; CI, confidence interval.

	HR (95% CI)	*P*
Variable		
LEF1 expression, LEF1^high^ vs LEF1^low^	3.7 (0.7- 18.9)	0.1
FLT3, ITD vs WT +TDK	0.8 (0.1- 3.8)	0.8
Sanz's score, L-I vs H	1.4 (0.3−6.4)	0.5

**Figure 3:**
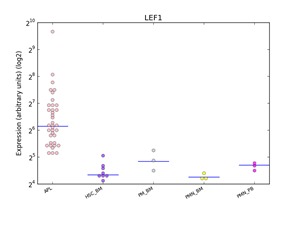
Distributions of *LEF1* expression in human haematopoiesis and in APL based on the HemaExplorer platform Each dot in the plot corresponds to the expression of *LEF1* in a microarray experiment. Horizontal lines represent the median expression value for each class of cells. Expression is given on the y-axis on a log2 scale. HSC_BM indicates hematopoietic stem cells from bone marrow; PM_BM, Promyelocytes from bone marrow; PMN_BM, Polymorphonuclear cells from bone marrow; PMN_PB, Polymorphonuclear cells from peripheral blood.

### *In silico* analysis of *LEF1* expression in APL

**Figure 4:**
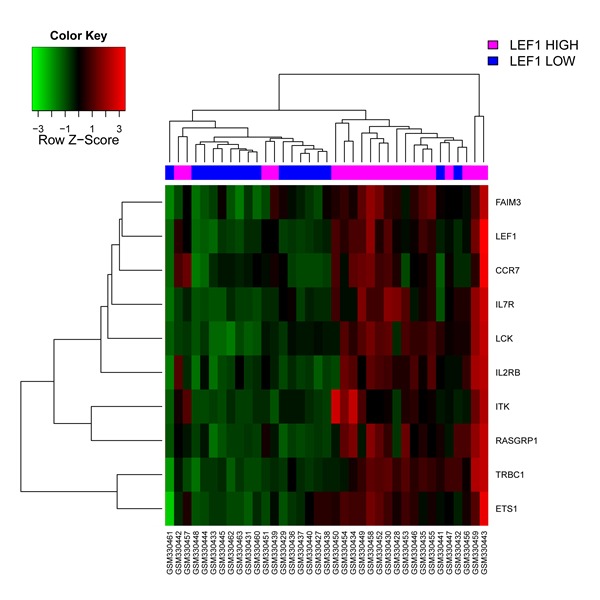
*In silico* analysis of *LEF1* expression in APL Heatmap image of the 9 differentially expressed genes associated with higher *LEF1* expression levels. Each column represents 1 of the 37 APL patients.

Using the HemaExplorer platform we observed that the *LEF1* gene expression median value was higher than in human physiological hematopoiesis (Figure 3). *In silico* analysis of the differential expression of the *LEF1* gene in APL identified 9 differentially expressed, up-modulated genes (*ETS1, FAIM3, CCR7, IL7R, LCK, IL2RB, ITK, RASGRP1, TRBC1*), associated with a high expression of *LEF1* (Figure 4); GO analysis revealed that the majority of these genes is involved in the regulation of apoptosis (*FAIM3, IL2RB, LCK, ETS1*).

**Figure 5:**
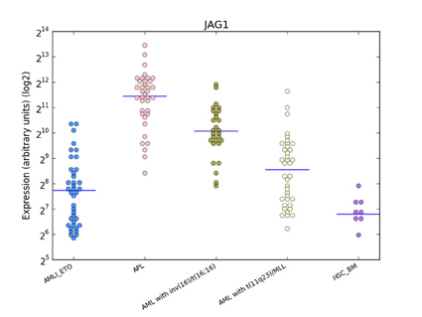
Distribution of JAG1 expression in human haematopoiesis and in AML based on the HemaExplorer platform Each dot in the plot corresponds to the JAG1 expression in a microarray experiment. Horizontal lines represent the median expression value for each class of cells. Expression is given on the y-axis on a log2 scale. HSC_BM indicates hematopoietic stem cells from bone marrow; PM_BM, promyelocytes from bone marrow; AMLI_ETO, AML with t(8;21); APL, AML with t(15;17); AML with inv(16)/t(16;16), AML with inv(16)/t(16;16); AML with t(11q23)/MLL, AML with t(11q23)/MLL.

**Figure 6:**
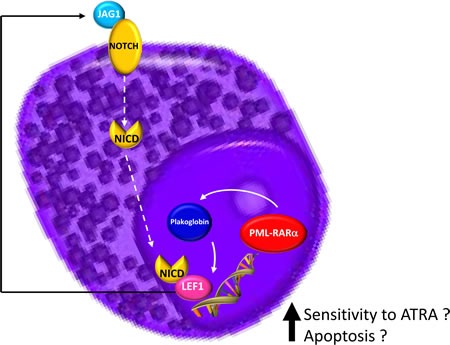
Schematic model summarizing the hypothesis at the basis of *LEF1* gene overexpression in APL

## DISCUSSION

To our knowledge, this is the first study to examine *LEF1* expression in a large cohort of APL patients and its correlation with clinical features and outcome. The association of LEF1^high^ status with a longer OS was confirmed in multivariate analyses adjusting for the most important prognostic factors in APL, such as age, *FLT3* status and Sanz score. This fact indicates that *LEF1* gene expression analysis is capable of discriminating APL patients with a poor outcome. In a recent paper analyzing 17 APL cases it was reported that patients with *PML-RARα* or *AML1-ETO* fusion genes had higher *LEF1* expression levels compared with AML cases without these translocations [19]. No data analysis was performed on the association between *LEF1* expression and clinical or biological features. The survival rate of APL elderly patients (>60 y.rs) is still controversial. While the European APL Group (EAG) and GIMEMA demonstrated that the survival rate of elderly APL was lower than that of younger patients [20,21], the PETHEMA group reported no significant difference [22]. Moreover, recently the Japan Adult Leukemia Study Group (JALSG) demonstrated that elderly APL patients were more prone to develop complications, which resulted in a lower OS [23]. Such evidence prompted us firstly to analyze the associations between *LEF1* gene expression and survival in the entire cohort and then to perform survival analysis by age (< 60 and > 60 y.rs). In both cases, the analysis showed that the LEF1^high^ group had a better outcome in terms of OS, revealing that LEF1^high^ status was a favorable prognostic marker in both age groups. Concerning patients younger than 60 years, two points should be highlighted: the first one is that these data were confirmed by multivariate analysis; secondly, the worst OS in the LEF1^low^ group cannot be explained by the association with ED, as only two cases of ED were observed in the younger than 60 years group.

Moreover, a higher proportion of patients in the LEF1^low^ group died before reaching first CR due to severe bleeding/infections and/or differentiation syndrome. It has been reported that these events are influenced mainly by older age, *FLT3*-ITD mutation status, WBC and platelet count at diagnosis [24-26]. According to these parameters, among the 9 patients who died early, 7 (77.7%) were classified as high risk and elderly, and 3 (33.3%) were *FLT3*-ITD positive. Of note, all patients belonged to the LEF1^low^ group. Therefore, our data indicate that *LEF1* gene expression, related to the Sanz score, age and the *FLT3*-ITD mutation, may be involved in the biological processes that underlie the prompt response to treatment, and that patients with low *LEF1* expression showed a significantly poorer outcome. We found that *FLT3*-ITD mutations are associated with low *LEF1* expression. This finding is in agreement with previous data describing this association in cytogenetically normal AML patients [13]. The prognostic significance of the *FLT3*-ITD mutation in APL remains controversial, as conflicting results have so far been reported about the correlation between *FLT3* status and OS. In fact, while some studies reported no association with outcome [26], others reported a poor outcome for *FLT3*-ITD-positive APL patients [27-30]. It is noteworthy that in our multivariate analysis performed on the two different patient groups according to age, the presence of the *FLT3*-ITD mutation was not associated with differences in terms of OS.

Dysregulated Wnt signaling has been identified in primary AML blasts, where it has been associated with poor survival [31-33]. If we consider *LEF1* expression as an activator of the Wnt pathway how can we explain, from a biological point of view, the paradoxical association in APL patients of a higher *LEF1* gene expression with a better prognosis? A possible explanation for this is that *LEF1* gene expression in the APL context is not a mark of a deregulated Wnt signaling. It has been reported that the *PML-RARα* fusion gene (but also *PLZF-RARα* and *AML1-ETO*) can induce plakoglobin (γ-catenin) expression in cell lines as well as in primary patient samples, resulting in transcriptional activation of *LEF1* [18]. The recent discovery made during a study of the mechanisms at the basis of the differentiation of bulge stem cells is intriguing. Indeed, it has been observed that *LEF1* crosstalks with the Notch signaling pathway, as *JAG1* is its downstream target [34]. This information is particularly relevant because it is known that *JAG1* is more strongly expressed in APL than in other AML subtypes [35] and that it is rapidly downregulated by ATRA treatment of NB4 cells and primary APL blasts [36,37]. *JAG1* upregulation in APL was also confirmed by our bioinformatics analysis (Figure 5). Moreover, recent findings support the hypothesis that Notch signaling is important in the pathogenesis of APL. In fact, bioinformatics analysis showed a Notch signature in both human APL and in mouse model cells, and experiments revealed that Notch inhibition blocked the enhanced self renewal in a pre-leukemic *PML-RARα* murine model [38]. Therefore, these data suggest that Notch signaling is a key downstream target of *PML-RARα*. Further evidence of the relationship between *LEF1* and Notch signaling is the finding that the Notch intracellular domain (NICD) has been identified as a coactivator of *LEF1*; the effects of Notch on *LEF1* activity are direct and not due to modulation of components of the Wnt signaling cascade [8]. Taken together, these data allow us to hypothesize a *LEF1* pathogenetic role in the context of APL (Figure 6).

Our in silico analysis revealed that LEF1^high^ status is characterized by an upregulation of genes that are differentially expressed in this group of patients, mostly linked to B-T cell function. It is noteworthy that 6 (*CCR7, IL7R, LCK, IL2RB, ITK, RASGRP1*) of the 9 genes were included among the signature of the 200 genes showing the strongest absolute correlation with *LEF1* expression levels in cytogenetically normal AML [13]. GO analysis showed that some of them are involved in apoptosis regulation mechanisms. This fact might explain the association between a high *LEF1* gene expression and lower WBC count. The *ETS1* gene has been described to contribute to human granulocytic differentiation. During the ATRA-induced granulocytic differentiation process in human NB4 promyelocytic and HL60 myeloblastic leukemia cell lines, the Ets-1 oncogenic protein is both down-regulated and inactivated; on the other hand, *ETS1* overexpression induces apoptosis [39].

Our data suggest that *LEF1* plays a role in APL but this circumstance is probably linked to stem cell aging. Unlike other forms of AML, APL is less frequently diagnosed in the elderly, indeed the median age at presentation is usually 40-45 years [40]. The observation in our study that *LEF1* overexpression is related with age suggests that the mechanisms underlying the APL pathogenesis may be different and age-related.

In conclusion, our study has shown that *LEF1* expression is a strong independent OS prognostic factor in APL; *LEF1* expression was measured by qRT-PCR, a routine technique in most diagnostic laboratories and therefore easy to use in clinical applications. It could therefore be useful to improve risk stratification and to develop better tailored treatment strategies in APL patients affected by *LEF1* low expression [41]. The observation, provided by in silico gene expression analysis, that *LEF1* expression is associated with biologic changes, mostly in terms of apoptosis regulation, will need to be experimentally confirmed, as well as the mechanisms regulating *LEF1* and their role in the pathogenesis of APL.

## METHODS

### Patients

One hundred and three consecutive patients with newly diagnosed APL were observed and treated with the AIDA-0493 [42] and AIDA-2000 [3] protocols at the Hematology Section, Bari University Hospital, between January 1996 and December 2012. The diagnosis was initially morphological and was confirmed in all cases by detection of the *PML-RARα* fusion gene as reported [43]. *LEF1* expression analysis by quantitative real-time PCR (qRT-PCR) was performed in 78 patients with sufficient available material (median age 45 years, range 16 to 88 years; 37 males and 41 females). The median follow-up time was 5.7 years for the entire cohort. All treatments were administered in accordance with the Declaration of Helsinki and approved by the institutional local review board, and all patients provided written informed consent. All 78 patients started induction treatment but 9 (11.5%) died within 30 days of admission (4 of them before definitive therapy could be instituted), 7 (8.9%) patients due to hemorrhagic/infective complications and 2 (2.5%) patients to the differentiation syndrome.

### Molecular analyses

Total RNA derived from bone marrow (BM) cells was reverse transcribed into cDNA using the QuantiTect reverse transcription kit (Qiagen, Chatsworth, CA, USA). Gene expression analysis was carried out by qRT-PCR experiments using the LightCycler 480 Probes Master mix on the LightCycler 480II (Roche Diagnostics, Indianapolis, IN, USA). All samples were run in triplicate as technical replicates; a pool of cDNA derived from BM cells of 5 healthy individuals was used as calibrator. *LEF1* expression was measured using a RealTime intron-spanning ready assay recognizing all 4 major human *LEF1* isoforms (assay ID 103366, Roche), and normalized to *GUSB* (β-glucuronidase) expression (assay ID 144221, Roche). Amplifications were carried out at 95°C for 10 min, followed by 45 cycles of 95°C (10 sec), 60°C (30 sec), 72°C (1 sec). Advanced relative quantification analysis was performed using the LightCycler 480 Software 1.5.1, based on the ∆∆Ct method.

*FLT3* (ITD and TKD) mutations were investigated on total BM RNA by allele specific oligonucleotide (ASO) - PCR and PCR followed by enzymatic digestion [44,45].

### Immunophenotypic analyses

Leukemic cell analysis was performed on bone marrow cells by standard immunofluorescence methods using monoclonal antibodies directed against CD2, CD3, CD4, CD5, CD7, CD8, CD10, CD11b, CD13, CD14, CD15, CD16, CD19, CD33, CD34, CD45, CD56, CD117, and HLA-DR (Becton Dickinson, Milan, Italy). All cases were studied by direct immunofluorescence. Flow cytometric analysis was performed on a FACSCanto™ II flow cytometer (Becton Dickinson Immunocytometry System, Mountain View, CA, USA). A sample was considered antigen-positive if ≥ 20% of the leukemic cells reacted with a particular monoclonal antibody.

### Statistical Analyses

APL samples were dichotomized at the median value and divided into two expression groups: a low *LEF1* group (LEF1^low^) with *LEF1* values below the median value (< 2.1 fold change) and a high *LEF1* group (LEF1^high^) with *LEF1* values above the median value (> 2.1 fold change). Clinical and biological features between groups were compared using the Fisher exact test for categorical data and the nonparametric Mann-Whitney *U* test for continuous variables. A *p* value <0.05 was considered significant. Survival curves were calculated by the Kaplan-Meier method with log-rank comparing differences between survival curves. Overall survival (OS) endpoints, measured from the date of diagnosis, were dead or alive at last follow-up. Relapse-free survival (RFS) was counted from the achievement of documented complete remission (CR) until relapse or death due to any cause, both considered uncensored events. The cumulative incidence of relapse (CIR) was estimated with the use of the proper nonparametric estimator, and between-group comparisons were performed with Gray's K-sample test [46,47]. Multivariable Cox proportional hazards models were used to study factors (*LEF1* expression, *FLT3* mutation status, age and relapse risk grade) associated with survival endpoints; no variable selection technique was used, and all variables remained in the multivariable model. Statistical analyses were carried out using GraphPad Prism version 6.01 for Windows (GraphPad Software, San Diego, CA) and XLSTAT version 2013.4.05 (Addinsoft™).

### *In silico* analysis of the biological role of *LEF1* in APL

We examined *LEF1* and Notch ligand Jagged-1 (*JAG1)* gene expression in human hematopoiesis and in APL using the HemaExplorer platform [48]. To evaluate the biological impact of *LEF1* differential expression in APL, we downloaded raw data from the publicly available dataset GSE13159 (Affymetrix HG-U133_Plus_2, available on the Gene Expression Omnibus,GEO, repository, http://www.ncbi.nlm.nih.gov/geo/); the dataset reported gene expression and clinical annotated data of 37 APL patients [49]. Raw gene expression data of APL patients were analyzed using R statistical language version 3.0.1 (URL http://www.r-project.org/). All samples were normalized and summarized with the Robust Multichip Average (RMA) normalization method. The mean intensity of the three probe sets for *LEF1* was calculated, and the one with the highest mean, 221558_s_at, was used to measure *LEF1* expression levels and to classify the samples into two groups: low or high *LEF1* expression, on the basis of the median expression value. Filtering was performed according to genes variability, and probe sets with an InterQuartile Range (IQR) <0.5 and without the associated gene symbol were excluded. To collapse and convert probe sets level expression data to gene-level, the probe set with the highest mean intensity for each gene was considered. After filtering and collapsing, 9990 probe sets were detected. Empirical Bayes t-test for Class Comparison analysis, imposing a False Discovery Rate (FDR) of <0.05 (p value adjustment method= Benjamini–Hochberg) identified the differentially expressed and up-modulated genes associated with a high expression of *LEF1*. Gene Ontology (GO) analysis was performed using the DAVID web server (http://david.abcc.ncifcrf.gov/) [50]. Only GO terms with a p-value < 0.01 were considered enriched and shown.

## Supplementary Figure


