# Supplementary material for: Lymphoid enhancer binding factor-1 (LEF1) expression as a prognostic factor in adult acute promyelocytic leukemia
Source: Oncotarget. 2013 Dec 19;5(3):649–58. doi: 10.18632/oncotarget.1619 (PMC3996670; doi:10.18632/oncotarget.1619)
Supplement: Supplementary file 1 [file oncotarget-05-0649-s001.pdf]

Lymphoid Enhancer Binding Factor-1 (*LEF1*) Expression as a Prognostic Factor in Adult Acute Promyelocytic Leukemia - Albano et al

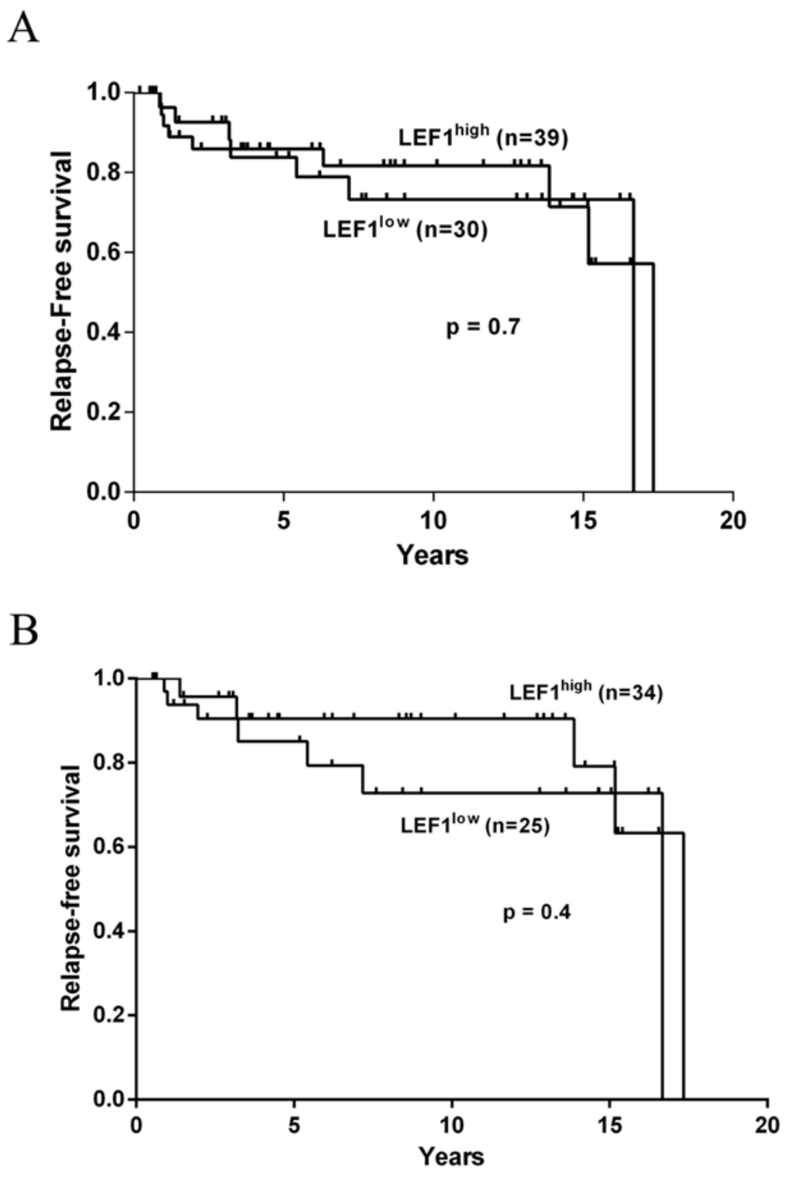

Supplementary Figure S1: Relapse-free survival analysis of APL patients according to *LEF1* expression. (A) Entire cohort of patients (B) Patients older than 60 years.
